# Identification of serum MiRNAs as candidate biomarkers for non-small cell lung cancer diagnosis

**DOI:** 10.1186/s12890-022-02267-6

**Published:** 2022-12-16

**Authors:** Xintong Zhang, Jinjing Tan, Yan Chen, Shang Ma, Wanqiu Bai, Yanjing Peng, Guangli Shi

**Affiliations:** 1 Department of Clinical Laboratory, Beijing Chest Hospital, Capital Medical University, Beijing, China; 2 Beijing Tuberculosis Thoracic Tumor Institute, Beijing, 101149 China; 3 Cancer Research Center, Beijing Chest Hospital, Capital Medical University, Beijing, China

**Keywords:** Non-small cell lung cancer, microRNA, Biomarkers, Diagnosis, miR-3149, miR-4769.3p

## Abstract

**Background:**

Lung cancer is one of the most common solid tumors worldwide and the leading cause of cancer-associated death. Non-small cell lung cancer (NSCLC) is accounts for approximately 85% of all the lung cancers and lung squamous carcinoma (SCC) and adenocarcinoma (ADC) are the main subtypes of NSCLC. Early diagnose using serum biomarkers could improve the overall survival of patients. In this study, we aimed to identify miRNAs from serum with clinical utility in the diagnosis of NSCLC.

**Methods:**

Ten patients with SCC, ten patients with ADC and five noncancerous individuals were enrolled in the screening cohort. miRNA expression levels in serum were measured by microarray analysis. Candidate miRNAs were validated by real-time quantitative polymerase chain reaction analysis in a validation cohort of 78 NSCLC patients and 44 noncancerous individuals. Receiver operating characteristic curves were used to assess the diagnostic performance of serum miRNAs for NSCLC. Logistic regression was used to evaluate the diagnostic value of the combination of markers.

**Results:**

Six candidate miRNAs were differentially expressed between NSCLC patients and noncancerous individuals in the screening set (fold change > 2, *p* < 0.05). Among them, expression levels of miR-3149 and miR-4769.3p were confirmed to be significantly increased in tumor serum in the validation set. The area under the curve values of miR-3149 and miR-4769.3p in distinguishing NSCLC patients from noncancerous controls were 0.830 and 0.735, respectively. When combined with tumor markers CEA and Cyfra21-1, the joint diagnostic model increased the area under the curve to 0.898.

**Conclusion:**

Serum miRNAs miR-3149 and miR-4769.3p were up-regulated in NSCLC and may be potential biomarkers for early diagnosis of lung cancer.

**Supplementary Information:**

The online version contains supplementary material available at 10.1186/s12890-022-02267-6.

## Introduction

Lung cancer is one of the most common solid tumors worldwide and the leading cause of cancer-associated death [1, 2]. Non-small cell lung cancer (NSCLC) accounts for approximately 85% of lung cancer cases, and the two most common histological subtypes are lung squamous carcinoma (SCC) and lung adenocarcinoma (ADC). The 5-year survival rate of NSCLC varies from 4 to 17% depending on stage and regional differences [3]. The stage of the disease at diagnosis has a strong impact on survival. The 2013–2017 cancer statistics data for England showed that the one-year net survival for lung cancer was the highest for patients diagnosed at Stage 1, and the lowest for those diagnosed at Stage 4. Approximately 88% of patients diagnosed at Stage 1 survived their disease for at least one year compared with 19% of patients diagnosed at Stage 4 [4]. Early diagnosis is crucial for lung cancer patients and can significantly improve five-year survival.

Low-dose computed tomography screening is recommended for early diagnosis of lung cancer and can reduce the mortality of lung cancer patients by 20% [5]. However, the application of low-dose computed tomography is limited by high costs and radiation damage [6]. Liquid biopsy, including serological testing, is still the most promising approach for in vitro diagnosis because of its advantages as a non-invasive method and its cost-effectiveness. Several serum biomarkers, which are abnormally expressed in tumors, are currently used in clinical practice, such as carcinoembryonic antigen (CEA) [7] and cytokeratin 19 fragment antigen 21-1 (Cyfra21-1) [8]. However, the sensitivity and specificity of these biomarkers are far from meeting clinical needs [9].

MicroRNAs (miRNAs) are a class of small noncoding RNAs that are approximately 18–22 nucleotides in length. MiRNAs participates in a majority of cancer related biological processes including cell proliferation, metastasis and drug resistance [10–12]. Unlike mRNAs and proteins, circulating miRNAs could existed stably in the serum [13]. Recent studies suggested that circulating miRNAs were either released by dead cells or secreted by cells in purpose of signaling [11]. Abnormally expressed circulating miRNAs may reflect the health state of the body. Thus, serum miRNAs may be potential tumor biomarkers in cancer diagnosis and prognosis.

In this study, we aimed to identify and verify the differentially expressed miRNAs (DEmiRNAs) in serum collected from NSCLC patients and noncancerous controls using microarray profiling and real-time fluorescent quantitative PCR (RT-qPCR). Our analyses identified several potential biomarkers for NSCLC early diagnosis.

## Materials and methods

### Study population

We retrospectively selected serum samples from patients who were newly diagnosed with NSCLC at Beijing Chest Hospital from 2019 to 2021. The NSCLC patients were diagnosed based on X-ray, computed tomograpgy (CT) and biopsy according to the World Health Organization’s Classification of Tumors of the Lung. Tumor stage was identified using the the 8th Edition TNM classification for lung cancer. Matched healthy individual were recruited from staff volunteers as noncancerous controls (NCs). Ten SCC patients, ten ADC patients and five NCs were randomly selected and grouped into the screening set, the remaining 78 NSCLC patients and 44 NCs were grouped into the validation group. The clinical and pathological information of all study participants including age, gender, smoking habit and tumor stage are provided in Tables 1 and 2. This study was approved by the Research Ethics Committee in Beijing Chest Hospital.

**Table 1:** Characteristic of the screening cohort

	SCC patients	ADC patients	Healthy control	*p* value
Total	10	10	5	
Gender				0.160^a^
Male	8	5	3	0.560^b^
Female	2	5	2	1.000^c^
Age	49–69	47–67	50–59	
Tumor stage				0.162^a^
I	3	4		
II	3	0		
III	0	2		
IV	4	4		

Statistic tests are performed on ^a^SCC versus ADC; ^b^SCC versus Healthy control; ^c^ADC versus Healthy control. * represents *p* < 0.05

**Table 2:** Characteristic of the validation cohort

	SCC patients	ADC patients	Healthy control	*p* value (SCC/ADC)
Total	39	39	44	
Gender				0.002^a^*
Male	35	23	21	< 0.001^b^*
Female	4	16	23	0.380^c^
Age	47–85	31–84	29–58	
Tumor stage				0.151^a^
I	3	9		
II	5	2		
III	10	6		
IV	21	22		

Statistic tests are performed on ^a^SCC versus ADC; ^b^SCC versus Healthy control; ^c^ADC versus Healthy control. *represents *p* < 0.05

### Sample processing and miRNA isolation

Peripheral blood from each participant was collected and processed for serum extraction. Samples were centrifuged at 3000 g for 10 min at room temperature. Serum was transferred to new EP tubes and stored at − 80 °C for future analysis.

For microarray analysis, total RNAs were extracted using TRIzol reagent (Invitrogen, Carlsbad, CA, USA) following the manufacturer’s instructions. miRNA labeling and hybridization were conducted with the miRNA Complete Labeling and Hyb Kit (Agilent Technologies, Santa Clara, CA, USA). For samples in the validation set, miRNAs were isolated from serum using the MiRNeasy Serum/Plasma kit (Qiagen, Valencia, CA, USA). RNA sample quality tests including RNA purity, total amount and integrity tests were performed. RNA purity was checked by the ratio of absorbance at 260 nm and 280 nm, a ratio of above 1.9 was accepted. The total RNA amount of each sample was at least 1 μg. RNA integrity was checked by agarose gel electrophoresis.

### miRNA microarray scanning and data analysis

The miRNAs from serum samples were hybridized with a Human miRNA microarray (Release 21.0, 8 × 60 K, Agilent Technologies), which was performed by Capitalbio Technology Corporation (Beijing, China). Agilent Feature Extraction (v10.7) software was used to read the original fluorescence intensity of the chip image and extract data from each probe. Agilent GeneSpring software was used to process data normalization and differentially expression analysis. miRNAs with significant differential expression were defined as those with fold change ≥ 2 and *p* < 0.05.

### miRNA reverse transcription and qPCR

First strand cDNA was synthesized by adding a poly-A tail of pre- and mature form of miRNA and reverse transcription was performed by RT-PCR (TransScript® miRNA First-Strand cDNA Synthesis SuperMix, TransGen Biotech, Beijing, China). Real-time quantitative PCR (qPCR) was carried out on an Applied BioSystems 7500 thermocycler using SYBR® select master mix (Applied BioSystems, Carlsbad, CA, USA). The qPCR reaction conditions were as follows: 95 °C for 10 min, followed by 40 cycles of 95 °C for 15 s and 60 °C for 1 min. PCR reactions were performed in duplicate for each sample. The housekeeping gene U6 was set as an internal reference. A random selected sample was used as an external reference. The relative miRNA expression was calculated using the equation 2^−∆∆CT^, in which ∆CT = cycle threshold (CT) of miRNA—CT of U6 gene in one sample and ∆∆CT = mean of ∆CT of test sample—mean of ∆CT of control sample. Three technical replicates were set for each sample. We conducted quality control on CT values to ensure that the standard deviation (SD) of CT values did not exceed 0.05. The means of the CT value were used to calculate ∆CT.

### Serum CEA and Cyfra21-1 detection

The levels of serum CEA and CYFRA21-1 were measured by an automatic flow fluorescence immunoanalyzer (model: tesmi) (Shanghai toujing biotechnology company, Shanghai, China). The detection process was performed following the detection kit protocol and standard operating procedures.

### GO and KEGG pathway analysis of miRNAs target genes

We performed prediction of target genes of significant DEmiRNAs using 12 databases: Targetscan [14], RNAhybrid [15], Pictar2 [16], miRNAMap [17], miRDB [18], miRWalk [19], MicroT v4 [20], miRanda [21], mirbridge [22], miRMap [23], PITA [24] and RNA22 [25]. Target genes that were predicted by at least seven databases were accepted for further analysis. GO annotation analysis and KEGG pathway [26–28] analysis were conducted on the target genes using R (version 3.6.5) package Cluster Profiler [29]. A *p* value < 0.05 was regarded as statistically significant.

### Protein–protein interaction (PPI) network analysis

The PPI network was constructed with the top 200 target genes using the online tool STRING (https://string-db.org/). We downloaded the interaction information and optimized the PPI network with Cytoscape software (v3.9.1) for better visualization.

### Statistical analysis

Data analysis was performed using R (version 3.6.5) and the statistical software GraphPad Prism8, (GraphPad Software Inc., CA, USA). The Pearson chi square test or Fisher’s exact test was used to analyze categorical variables. Student’s t-test was used to compare continuous variables. The values of miRNA expressions that were not normally distributed were analyzed by Mann–Whitney test. A *p* value less than 0.05 was regarded as statistically significant. ROC curves were established to analyze the diagnostic effects of serum miRNAs and the AUC calculated their specificity and sensitivity in the diagnosis of NSCLC. To predict the diagnostic efficacy of combinations of multiple biomarkers, regression models using binary logistic regression method were used. ROC analysis was conducted with R package pROC [30].

## Results

### Screening for DEmiRNAs in serum from NSCLC patients

To discover the serum miRNA biomarkers for lung cancer early diagnosis,a total of 98 NSCLC and 49 healthy serum samples were collected in the study. The serum samples were randomly divided into the screening set and validation set. The screening group consisted of 10 SCC samples, 10 ADC samples and 5 NC samples. Table 1 summarizes the demographic and clinical characteristics of the population in the screening set, including age, gender, histological type and tumor stage. There was a slight gender difference between SCC and ADC patients, with a higher proportion of male patients in the SCC group. This feature is consistent with observations in clinical practice. This difference was not statistically significant in the screening cohort (*p* > 0.05). There was no significant difference in tumor stage distribution between SCC and ADC groups in the screening cohort (*p* = 0.162).

**Fig. 1:**
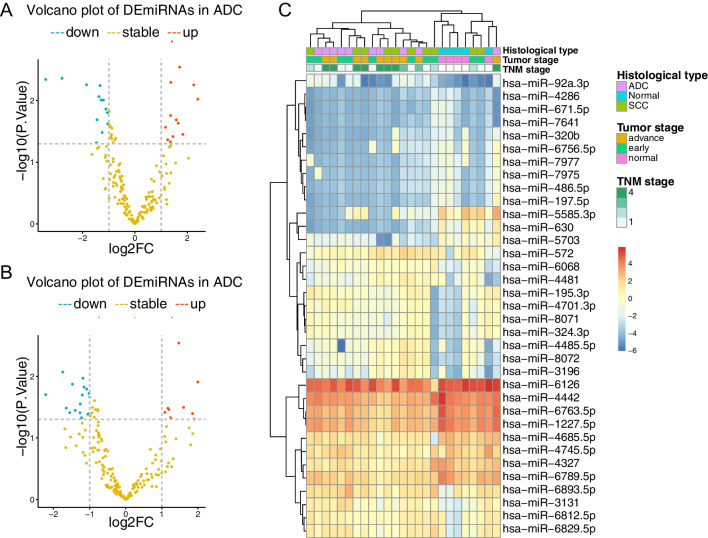
Screening for differentially expressed miRNAs (DEmiRNAs) between tumor and NC sera. Volcano plot of DEmiRNAs in **A** SCC sera and **B** ADC sera. Dashed lines indicate the screening threshold, which is fold change ≥ 2 and *p* < 0.05. **C** The top 20% DEmiRNAs ranked by fold change were selected for heatmap analysis. K-means was used to perform unsupervised hierarchical clustering on miRNA expressions

**Fig. 2:**
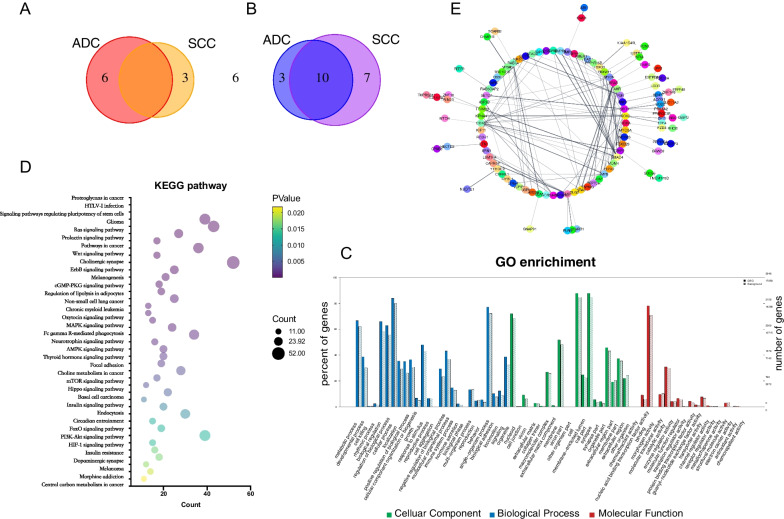
Exploring differentially expressed miRNAs (DEmiRNAs). Intersection of significantly DEmiRNAs between **A** up-regulated and **B** down-regulated miRNAs between SCC patients and ADC patients. **C** GO enrichment analysis of potential target genes regulated by DEmiRNAs. Enrichment categories are distinguished by color, blue is biological process (BP); green is cellular component (CC); red is molecular function (MF). **D** KEGG analysis of potential target genes regulated by DEmiRNAs was selected. The gene count is shown on the x-axis, and the pathway terms are on the y-axis; bubble size is gene count and bubble color reflects *p* value. **E** Potential target genes enriched in top 20 KEGG pathway was selected to performed protein–protein interaction (PPI) network analyisi. Line indicated interaction Between two proteins

Total RNAs were extracted from serum samples and DEmiRNAs were screened. A total of 2750 miRNAs was detected by each sample using the Agilent Human miRNA Microarray, release 21.0. The results revealed that 193 serum miRNAs were differentially expressed between tumor samples and NC samples (Fig. 1A, B). The expression pattern of DEmiRNAs could distinguish tumor from NC samples (Fig. 1C). Significant DEmiRNAs were defined as those with a *p* value less than 0.05 and more than two-fold change. In the ADC samples, 12 miRNAs were significantly up-regulated and 13 miRNAs were significantly down-regulated. In the SCC samples, 9 miRNAs were significantly up-regulated and 17 miRNAs were significantly down-regulated. We intersected the results from the ADC samples and the SCC samples and obtained 16 significant DEmiRNAs (Fig. 2A, B).

We next predicted the target genes that were regulated by the common significant DEmiRNAs. GO enrichment and KEGG pathway analysis was performed on these target genes to investigate the potential function of DEmiRNAs (Fig. 2C). The target genes were enriched in various biological processes such as metabolic process and cell killing. The KEGG pathway analysis suggested that the DEmiRNAs were envolved in pathways such as Ras signaling, Wnt signaling and MAPK signaling (Fig. 2D). We performed PPI analysis of the top predicted target genes. PPI network revealed that the hub genes were *KRAS*, *Sox2*, *SIRT1*, *SMAD4* and *CDK6* (Fig. 2E)*.*

**Fig. 3:**
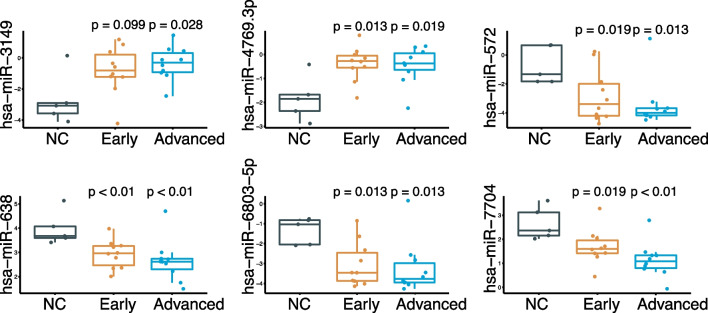
The expression of six candidate serum miRNAs (miR-3149, miR-4769.3p, miR-572, miR-638, miR-6803.5p and miR-7704) in screening samples. Mann–Whitney tests were performed to ascertain statistical significance between the expression levels across groups from early and advanced patients compared to controls

To explore biomarkers for early diagnosis, we selected the miRNAs whose serum level began to change in the early stage of disease and intensified as the disease progressesd. Six candidate miRNAs were selected for further validation. Among the six miRNAs, miR-3149 and miR-4769.3p were up-regulated in tumor serum, while miR-572, miR-638, miR-6803.5p and miR-7704 were down-regulated in tumor serum (Fig. 3).

### Validation of candidate miRNAs

We next performed validation analysis of the candidate miRNAs in the validation set including 78 NSCLC patients and 44 NCs. The demographic and clinical characteristics of the population in the validation set are summarized in Table 2. Similar to the screening cohort, a gender difference was observed between SCC patients and ADC patients, and between SCC patients and healthy controls. The gender difference in the validation cohort was statistics significant (*p* < 0.05). There was no significant difference in tumor stage distribution between SCC patients and ADC patients in the validation cohort (*p* = 0.151).

**Fig. 4:**
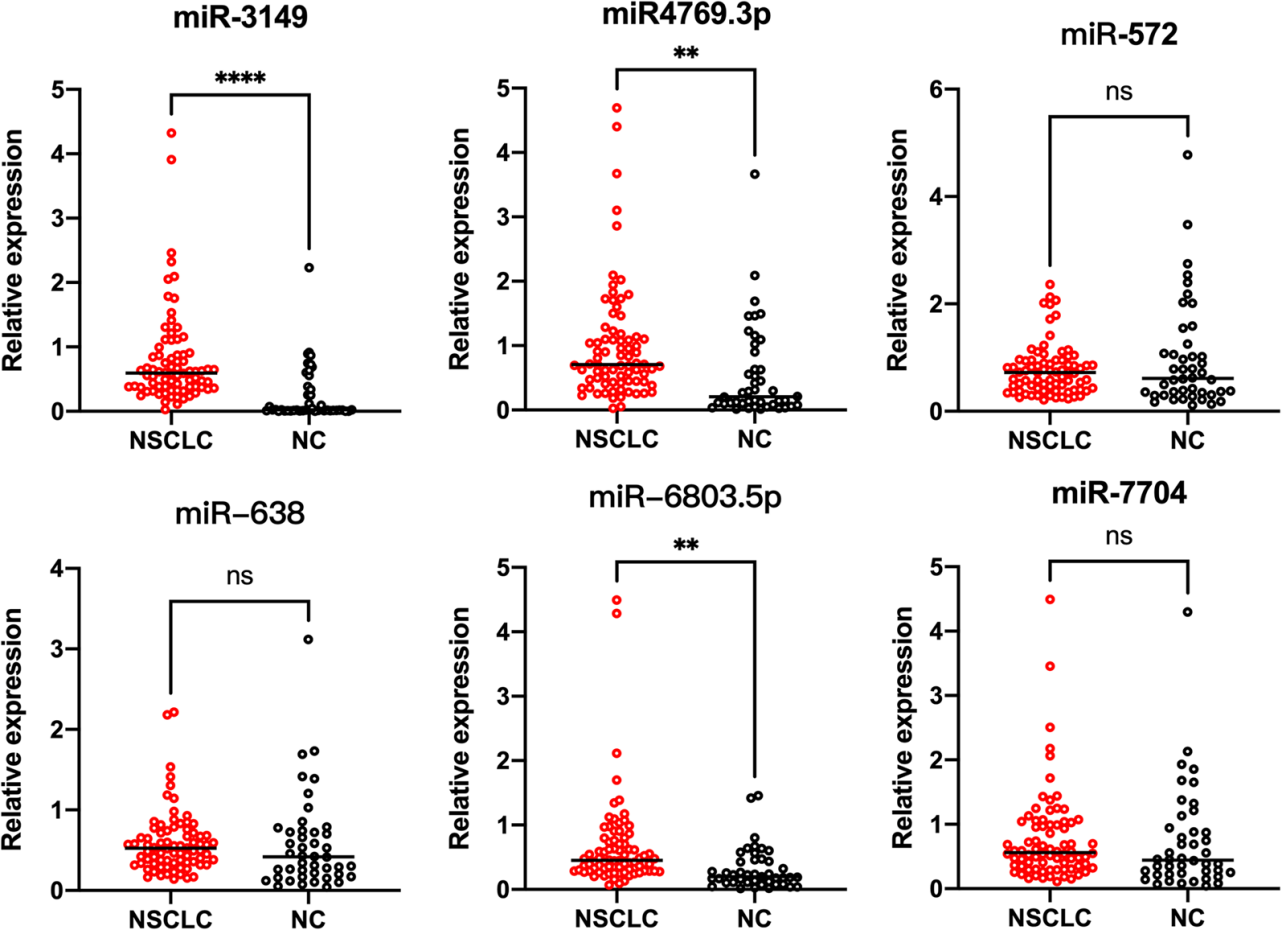
Serum expression levels of candidate miRNAs in validation set. The miRNA levels of miRNA-3149, miR-4769.3p, miR-6803, miR-572, miR-638 and miR-7704 were detected by RT-qPCR. Ct data were transformed to relative expression fold to reference sample. ** represents *p* < 0.01, **** represents *p* < 0.001, ns represents not significant

We extracted miRNAs from serum samples of the validation set, and the six candidate serum miRNAs obtained by initial screening were examined by RT-qPCR. As shown in Fig. 4, miR-3149 and miR-4769.3p were significantly up-regulated in NSCLC patients compared with NCs (*p* values were both less than 0.01 respectively). Interestingly, even though serum expression level of miR-6803.5p was significantly different between NSCLC patients and NCs, the changes were microarray and RT-qPCR results were in opposite directions. Therefore, miR-6803.5p was considerate as failed validation. The remaining three miRNAs (including miR-572, miR-683 and miR-7704) were also not verified. Their expression level in tumor serum in the validation group were not significantly lower than in NCs (*p* > 0.05).

We also performed subgroup analysis on serum miRNA expression levels in patients with different clinical characteristics. We compared the serum miRNA level between tumor patients with the two cancer subtypes (SCC and ADC) and compared serum miRNA levels between patients with early tumor stage (stage 1 and 2) and advanced tumor stage (stage 3 and 4) using nonparametric statistical methods. The serum level of miR-3149 and miR-4769.3p in NSCLC was not associated with gender, histological type and tumor stage (Table 3).

**Table 3:** Comparison of clinical characteristics with miRNAs expression in serum samples

Characteristics	Number	miR-3149			miR-4769.3p		
		mean (range)	Z score	*p* value	mean (range)	Z score	*p* value
Disease status							
Cancer	78	0.596 (0.374–0.885)	6.051	< 0.001	0.711 (0.442–1.128)	4.329	< 0.001
Noncancerous	44	0.220 (0.116–0.374)			0.207 (0.088–0.834)		
Histological type							
SCC	40	0.632 (0.376–1.117)	1.13	0.259	0.788 (0.452–1.101)	0.58	0.562
ADC	38	0.492 (0.371–0.829)			0.662 (0.428–1.194)		
Tumor stage							
I + II	20	0.521 (0.386–0.780)	0.698	0.485	0.839 (0.622–1.217)	1.305	0.192
III + IV	58	0.616 (0.357–1.107)			0.681 (0.370–1.110)		

### Diagnostic efficacy of serum miR-3149 and miR-4769.3p

**Fig. 5:**
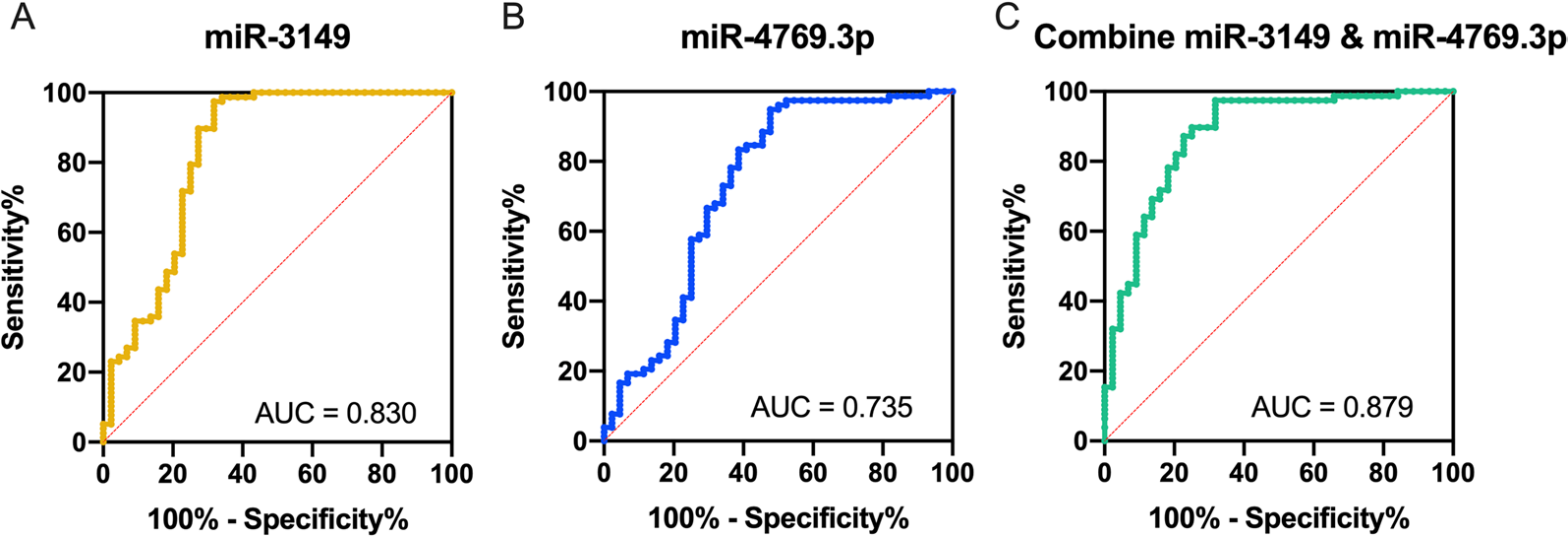
Receiver operator characteristic (ROC) curve analyses of **A** miR-3149, **B** miR-4769.3p and **C** the combination of these two miRNAs

miR-3149 and miR-4769.3p showed high efficiency in distinguishing NSCLC patients from NCs in the validation set, with AUC values of 0.830 and 0.735, respectively (Fig. 5A, B). We used Youden’s index to select an appropriate cutoff to calculate the sensitivity and selectivity of miRNAs in diagnosing NSCLC. At the cutoff of 0.131, miR-3149 diagnosed NSCLC with a sensitivity of 97.44% and the specificity of 68.18%. At the cutoff of 0.219, miR-4769.3p diagnosed NSCLC with a sensitivity of 94.87% and specificity of 52.25%. The combination of the two miRNAs showed a slightly higher AUC value of 0.879. At the cutoff of Youden’s index, the diagnosis sensitivity was 97.44% and the specificity was 68.18% (Fig. 5C).

The differential expression analysis of miRNAs and ROC analysis in the validation group were also performed with regard to cancer subtypes, ex. SCC versus control and ADC versus Control (Additional file 1: Fig. S1 and Additional file 2: Fig. S2). The results were similar to those described above for the NSCLC versus Control analyses. These results indicated that the diagnostic power of the serum biomarkers did not differ between tumor subtypes.

### Diagnostic efficacy of combination of multiple serum biomarkers

**Fig. 6:**
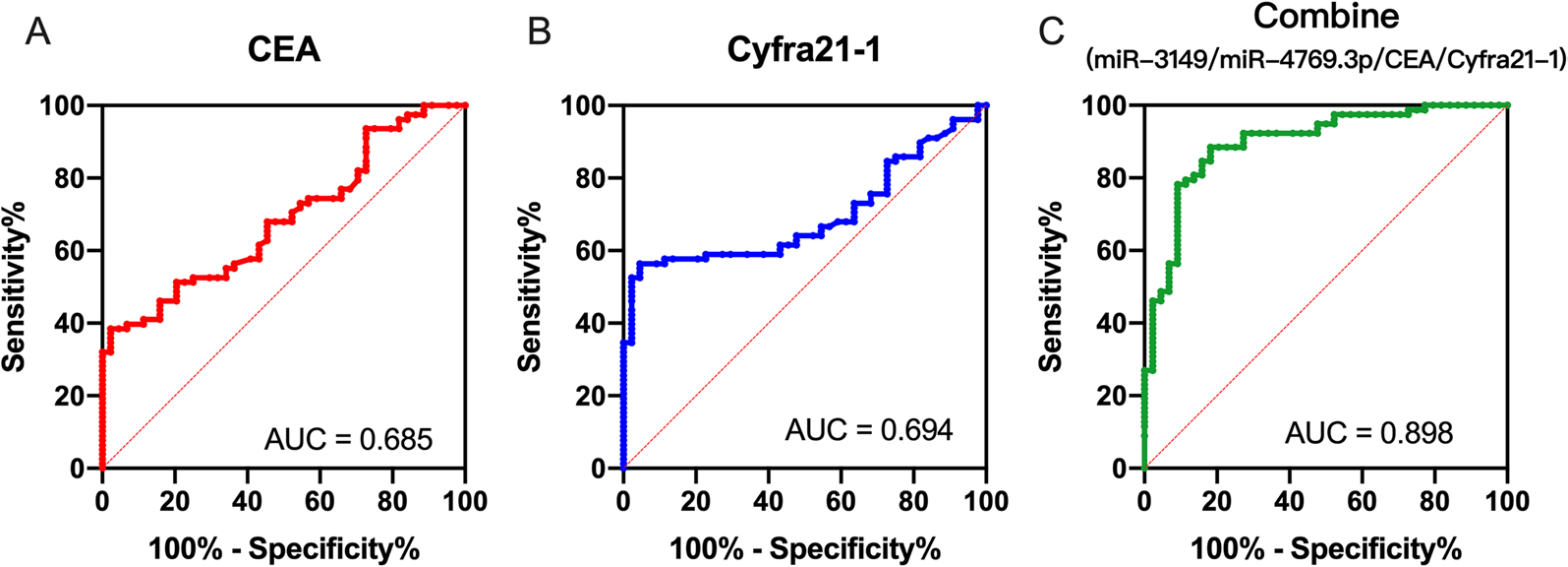
Receiver operator characteristic (ROC) curve analyses of **A** CEA, **B** Cyfra21-1 and **C** the combination of miRNAs, CEA and Cyfra21-1

CEA and Cyfra21-1 are commonly used tumor serum biomarkers in the clinical screening and early diagnosis of lung cancer. The serum concentrations of CEA and Cyfra21-1 in NSCLC patients and healthy individuals, as well as in different NSCLC subgroups are summarized in Table 4. The expressions of serum CEA and Cyfra21-1 were up-regulated in NSCLC patients and increased with tumor progression. Expression levels of CEA and Cyfra21-1 in patients with advanced tumor stage was higher in patients with early tumor stage (*p* < 0.05). Their expression was markedly different between SCC patients and ADC patients. CEA was significantly higher in ADC patients than in SCC patients (*p* = 0.016), while Cyfra21-1 showed the opposite trend (*p* = 0.039). ROC curves indicated that the diagnostic efficacies of CEA and Cyfra21-1 were moderate, with the AUC value at 0.685 and 0.694, respectively (Fig. 6A, B). When using the combination of the four indicators (CEA, Cyfra21-1, miR3149 and miR-4769.3p) to distinguished NSCLC patients from NC, the AUC of ROC curve was greatly improved to 0.898 (Fig. 6C). At the cutoff of the Youden’s index, the diagnosis sensitivity was 88.46% and the specificity was 81.82%.

**Table 4:** Comparison of clinical characteristics with CEA and Cyfra21-1 expression in serum samples

Characteristics	Number	CEA (ng/mL)			Cyfra21-1 (ng/mL)		
		mean (range)	Z score	*p* value	mean (range)	Z score	*p* value
Disease status							
Cancer	78	2.925 (1.237–8.607)	3.534	< 0.001	4.200 (1.307–11.48)	3.607	< 0.001
Noncancerous	44	1.650 (0.830–2.740)			2.280 (0.815–2.930)		
Histological type							
SCC	40	2.210 (1.195–3.567)	2.409	0.016	4.620 (1.975–17.94)	2.069	0.039
ADC	38	4.475 (1.560–16.39)			3.255 (1.032–7.590)		
Tumor stage							
I + II	20	1.685 (1.115–3.130)	2.552	0.011	1.435 (0.660–4.830)	2.718	0.007
III + IV	58	3.400 (1.587–10.85)			4.785 (2.052–14.69)

## Discussion

Lung cancer is the leading cause of cancer-related death in the worldwide [1, 2]. Diagnosis of lung cancer at late stage is the most important contributor to the high mortality of NSCLC. Several tumor serum biomarkers, such as CEA and Cyfra21-1, are currently used in clinical practice. However, the diagnostic efficacy of these markers is not satisfactory. Therefore, new methods or biomarkers for lung cancer diagnosis are urgently needed. Previous studies indicated that abnormally expressed miRNAs may be potential diagnostic biomarkers of NSCLC [31].

Circulating miRNAs are promising liquid biopsy targets that are tissue specific [32]. The tissue origin of lung SCC and lung ADC is different, which results in unique expression patterns of miRNAs. Therefore, we obtained the DEmiRNAs of SCC serum and ADC serum and identified intersecting DEmiRNAs as candidate miRNAs for validation. In order to discover novel biomarkers, we selected DEmiRNAs with high fold expression change and less reports for subsequent analysis.

After validation in another sample set, we demonstrated that the serum miR-3149 and miR-4769.3p levels were significantly increased in NSCLC patients and could helped distinguishing patients from NCs. Earlier studies focused only on its abnormally expression in acute coronary syndrome and as a potential biomarker for disease warning [33–35]. A recent study found that exosomal miR-3149 was down-regulated in plasma from gastric cancer patients [36]. Limited studies have been performed on miR-4769.3p and cancer. One study reported that miR-4769.3p was expressed at lower evels in serum of metastatic cervical squamous cell carcinoma patients compared with non-metastatic cervical squamous carcinoma patients. However, miR-4769.3p could not distinguish between cervical squamous cell carcinoma patients and healthy individuals [37]. The functions of miRNAs also need to be further explored.

We also detected the expression level of CEA and Cyfra21-1 in serum samples. Although serum CEA and Cyfra21-1 expression were significantly higher in tumor patients than in healthy individuals (Table 4), their performance was not ideal in ROC curve analysis (Fig. 5). In addition, CEA and Cyfra21-1 serum expression levels were different between the two subtypes of lung cancer. CEA was higher in ADC and Cyfra21-1 was higher in SCC. However, in the real-world situation, we can not predict the tumor subtype of the patients, so it is meaningless for a biomarker to have a high diagnostic efficiency for a certain tumor subtype. Using serum miRNAs either alone or in combination showed a higher AUC than CEA and Cyfra21-1. The combined use of miR-3149 and miR-4769.3p with CEA and Cyfra21-1 may greatly improve the diagnostic efficiency of lung cancer.

Our study has several limitations. First, relative expression levels were used in this study to evaluate the expression level of candidate miRNA markers among samples. The external reference in this study was a random serum sample of a patient. Such reference materials are unreproducible and are not conductive to subsequent experiments. A synthetic miRNA mimic as a standard or quantitative detection methods may be more suitable. Additionally, multicenter studies with larger sample sizes will be required to confirm the current findings.

## Conclusion

Serum miR-3149 and miR-4769.3p levels were significantly elevated in NSCLC patients compared to NCs. Serum miR-3149 and miR-4769.3p expression may be a promising biomarker early diagnosis of NSCLC.

## Supplementary Information

**Additional file 1: Fig. S1.** The expression levels and diagnostic efficacy of six candidate serum miRNAs in ADC from validation set. The miRNA levels of miRNA-3149, miR-4769.3p, miR-6803, miR-572, miR-638 and miR-7704 were detected by RT-qPCR. Ct data were transformed to relative expression fold to reference sample. ** represents p < 0.01, **** represents p < 0.001, ns represents not significant. Receiver operator characteristic (ROC) curve analyses of miR-3149, miR-4769.3p and miR-6803.5p, which were significantly increased or reduced in ADC patients.

**Additional file 2: Fig. S2.** The expression levels and diagnostic efficacy of six candidate serum miRNAs in SCC from validation set. The miRNA levels of miRNA-3149, miR-4769.3p, miR-6803, miR-572, miR-638 and miR-7704 were detected by RT-qPCR. Ct data were transformed to relative expression fold to reference sample. ** represents p < 0.01, **** represents p < 0.001, ns represents not significant. Receiver operator characteristic (ROC) curve analyses of miR-3149, miR-4769.3p and miR-6803.5p, which were significantly increased or reduced in SCC patients.

## Data Availability

The datasets used and analyzed during the current study are available from the corresponding author on reasonable request.

## Abbreviations

NSCLC: Non-small cell lung cancer
ADC: Adenocarcinoma
SCC: Squamous cell carcinoma
CEA: Carcinoembryonic antigen
Cyfra21-1: Cytokeratin 19 fragment antigen 21-1
LDCT: Low-dose computed tomography
RT-qPCR: Real-time fluorescent quantitative PCR
