# Supplementary figures and images for: Identification of serum MiRNAs as candidate biomarkers for non-small cell lung cancer diagnosis
Source: BMC Pulm Med. 2022 Dec 16;22:479. doi: 10.1186/s12890-022-02267-6 (PMC9756610; doi:10.1186/s12890-022-02267-6)

Cancer subtype : ADC

miR-3149

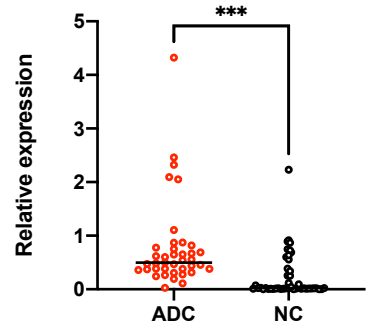

miR4769.3p

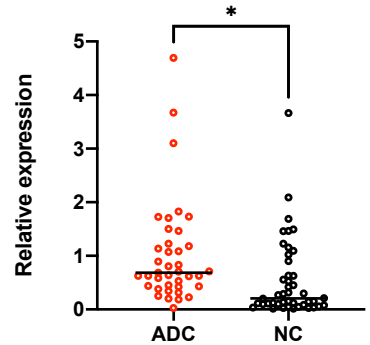

miR-6803.5p

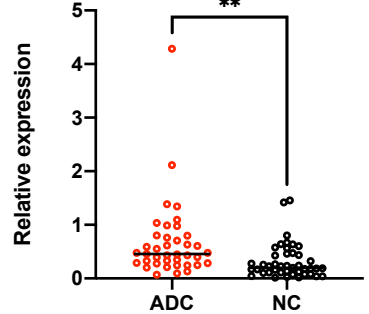

miR-3149

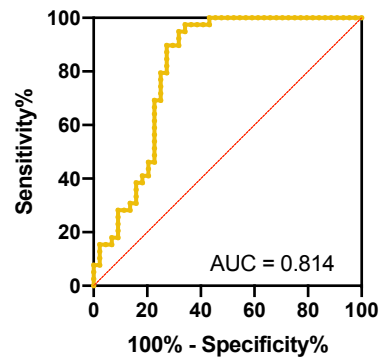

miR-4769.3p

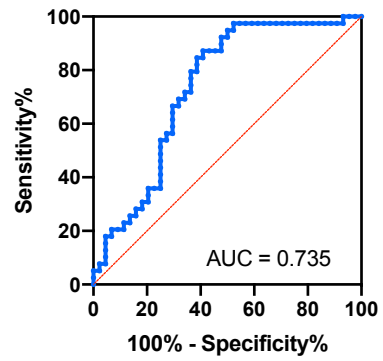

miR-6803.5p

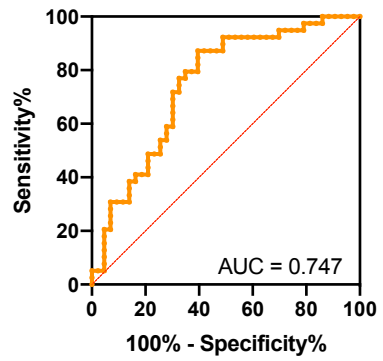

miR-572

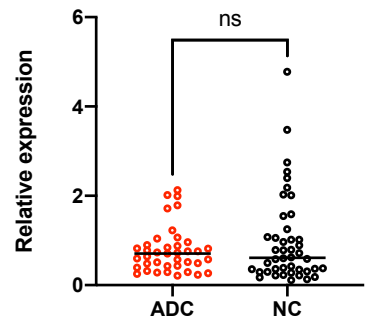

miR-638

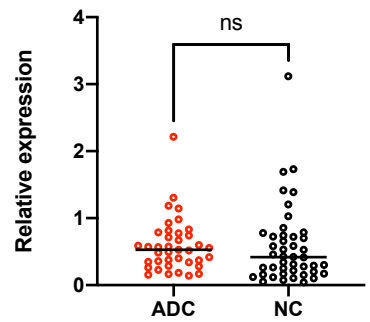

miR-7704

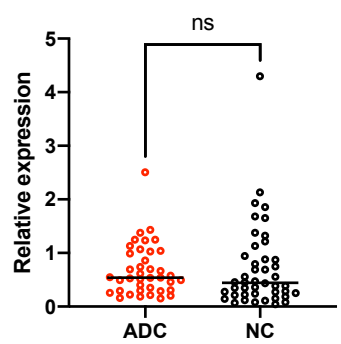

Supplement: Supplementary file 1 — Additional file 1: Fig. S1. The expression levels and diagnostic efficacy of six candidate serum miRNAs in ADC from validation set. The miRNA levels of miRNA-3149, miR-4769.3p, miR-6803, miR-572, miR-638 and miR-7704 were detected by RT-qPCR. Ct data were transformed to relative expression fold to reference sample. ** represents p < 0.01, **** represents p < 0.001, ns represents not significant. Receiver operator characteristic (ROC) curve analyses of miR-3149, miR-4769.3p and miR-6803.5p, which were significantly increased or reduced in ADC patients. [file 12890_2022_2267_MOESM1_ESM.pdf]

Cancer subtype : SCC

miR-3149

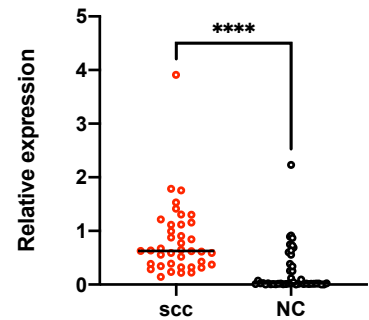

miR4769.3p

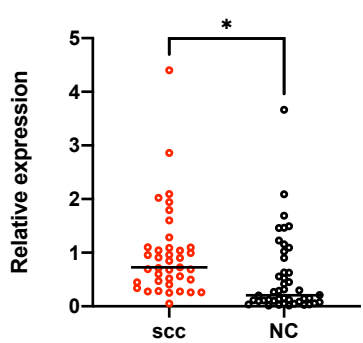

miR-6803.5p

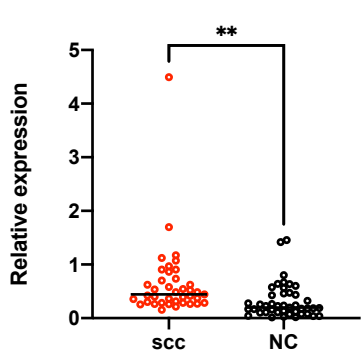

miR-3149

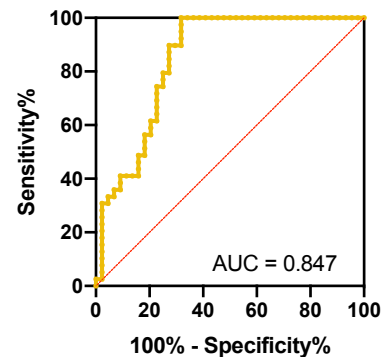

miR-4769.3p

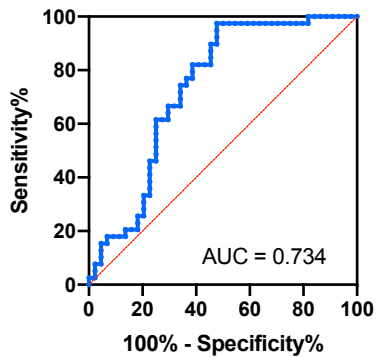

miR-6803.5p

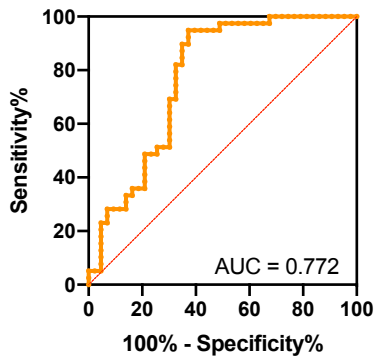

miR-572

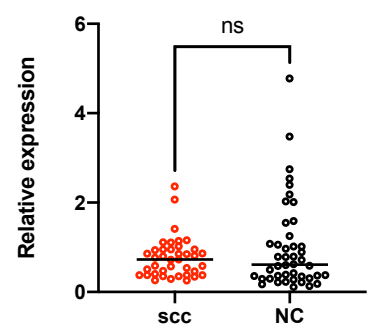

miR-638

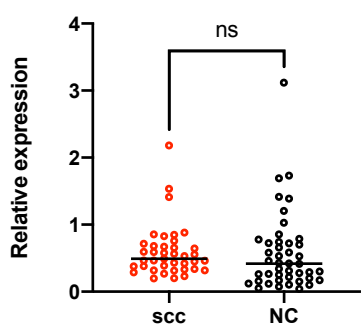

miR-7704

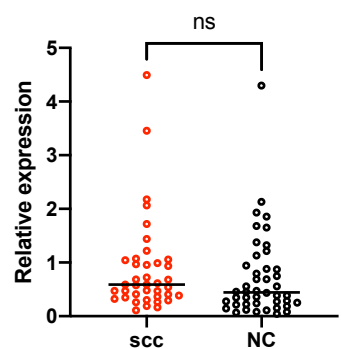

Supplement: Supplementary file 2 — Additional file 2: Fig. S2. The expression levels and diagnostic efficacy of six candidate serum miRNAs in SCC from validation set. The miRNA levels of miRNA-3149, miR-4769.3p, miR-6803, miR-572, miR-638 and miR-7704 were detected by RT-qPCR. Ct data were transformed to relative expression fold to reference sample. ** represents p < 0.01, **** represents p < 0.001, ns represents not significant. Receiver operator characteristic (ROC) curve analyses of miR-3149, miR-4769.3p and miR-6803.5p, which were significantly increased or reduced in SCC patients. [file 12890_2022_2267_MOESM2_ESM.pdf]
